# Clinical manifestations, outcomes, and antibody profile of Brazilian adult patients with dermatomyositis: a single-center longitudinal study

**DOI:** 10.1186/s42358-022-00276-x

**Published:** 2022-11-12

**Authors:** Natássia Cristina Carboni Truzzi, Leonardo Santos Hoff, Isabela Bruna Pires Borges, Fernando Henrique Carlos de Souza, Samuel Katsuyuki Shinjo

**Affiliations:** 1 Division of Rheumatology, Faculdade de Medicina FMUSP, Universidade de Sao Paulo, Av. Dr. Arnaldo, 455, 3º Andar, sala 3184 - Cerqueira César, Sao Paulo, SP CEP: 01246-903 Brazil; 2 Division of Rheumatology, Faculdade de Medicina, Universidade Potiguar (UnP), Natal, RN Brazil; 3 Division of Rheumatology, Hospital das Clinicas HCFMUSP, Universidade de Sao Paulo, Sao Paulo, SP Brazil

**Keywords:** Dermatomyositis, Autoantibodies, Longitudinal study, Outcome, Treatment

## Abstract

**Background:**

Currently, only a few retrospective cohort or cross-sectional studies have described the general characteristics of Brazilian patients with classical dermatomyositis (DM). In contrast, we aimed to longitudinally assess a large sample of these patients, and several myositis autoantibodies.

**Methods:**

This single-center longitudinal study included 91 Brazilian adults with defined DM (EULAR/ACR 2017) who underwent follow-up appointments in our tertiary center from 2012 to 2021. Myositis autoantibody analysis was performed using a commercial kit.

**Results:**

The mean age of the patients was 47.3 ± 15.4 years, with a predominance of female (67.0%) and White (81.3%) patients. As an initial treatment, 76.9% of the patients received methylprednisolone pulse therapy, 59.3% received intravenous immunoglobulin, and 54.9% received both drugs. The median follow-up duration was 44 months (interquartile 17–67) months. There were 92 severe episodes of infection, and neoplasms were identified in 20 patients (22.0%). Hypertension was identified in 46.2% of patients, whereas diabetes mellitus and myocardial infarction occurred in 19.8% and 4.4%, respectively. Nine patients died during the follow-up. At the last visit, one-third of the patients had disease activity, half had a complete clinical response, and one-fifth were in disease remission. In a univariate logistic regression, anti-aminoacyl-tRNA synthetase antibodies (n = 13) were associated with interstitial lung disease, “mechanic’s hands”, and anti-Ro-52, and had an inverse association with “V”-neck and “shawl” signs. Anti-MDA-5 (n = 10) were associated with male gender, digital ulcers, vasculitis, arthritis, anti-Ro-52, and active disease. Anti-Ro-52 (n = 26) were associated with “mechanics’ hands”, arthritis, interstitial lung disease, anti-tRNA synthetases, and anti-MDA-5. No association was found for anti-Mi-2 (n = 10).

**Conclusions:**

This study shows the general profile of a significant sample of Brazilian patients with DM as well as the association of some antibodies with clinical and laboratory manifestations of this myositis.

**Supplementary Information:**

The online version contains supplementary material available at 10.1186/s42358-022-00276-x.

## Introduction

Dermatomyositis (DM) is a systemic autoimmune myopathy characterized by skin involvement and predominant proximal limb muscle weakness [1]. Concerning cutaneous manifestations, patients with DM may show classic signs, such as heliotrope and papules/Gottron’s sign, whereas secondary cutaneous disorders include facial rash, “V-neck sign,” “shawl sign,” ulcers, vasculitis, calcinosis, and others. Moreover, manifestations in the joints, lungs, heart, and gastrointestinal tract may also be present [2, 3].

Myositis autoantibodies have been detected in up to 60% of patients with inflammatory myopathies. Those that are most relevant for DM are anti-Jo-1, anti-Mi-2, anti-MDA-5, anti-TIF1-γ, anti-SAE, and anti-NXP-2 [4, 5]. Detection of these autoantibodies allows for better characterization of DM’s phenotypic pattern of DM and establishes associations with clinical manifestations and prognosis [6–21]. The autoantibody profile and associations with DM manifestations vary according to the geographic area.

Only a few epidemiological studies of patients with DM have been conducted [6–23]. The majority of these studies were limited to cross-sectional [7, 9, 11, 16, 17] or retrospective studies [6, 12, 14, 15, 18, 20, 22], with a small number of patients with DM [13, 18, 23] or heterogeneous cohorts, consisting of patients with probable or possible DM diagnosis [9, 11, 13, 18, 23]. Furthermore, only five studies analyzing myositis-specific autoantibodies have been performed in a Brazilian population with DM [6, 7, 14, 15, 17].

Therefore, the present study aimed to longitudinally evaluate the clinical, laboratory, and evolutive profiles of a significant sample of Brazilian patients with a definitive DM diagnosis, as well as to analyze possible associations between myositis-specific autoantibodies and myositis-associated autoantibodies with the characteristics of this disease.

## Patients and methods

This was a longitudinal, single-center study. Adult patients with classic DM were assessed at the Inflammatory Myopathies Clinic of our tertiary service between January 2012 and July 2021. This study was approved by the local research ethics committee (CAAE 68523717.1.0000.0068) and all participants signed the term free and informed consent.

Only patients at least 18 years old and with definite DM per the 2017 European League Against Rheumatism/American College of Rheumatology (EULAR / ACR) criteria classification were included [24]. Patients with clinically amyopathic DM [25], overlap syndromes, and other inflammatory myopathies were excluded.

Demographic features, treatment, clinical, and laboratory data were obtained at the first and last medical visits, and any missing information was retrieved from the patient's files.

We collected demographic, clinical, laboratory, disease activity status, and therapeutic information using pre-standardized and parameterized information.

- Demographics: Age at disease diagnosis, sex, and ethnicity.
- Clinical manifestations: symptoms onset, disease duration, outpatient follow-up time, cutaneous involvement (heliotrope rash, Gottron’s papules/sign, facial rash, “mechanic’s hand,” ulcers, calcinosis, vasculitis, “V-neck sign,” “shawl sign,” and periungual hyperemia), Raynaud’s phenomenon, systemic manifestations (dysphagia, arthritis, dysphonia, dyspnea, and weight loss); limb muscle strength graded according to the Medical Research Council (MRC) classification [26].
- Complementary examinations: serum levels of muscle enzymes in blood samples [creatine phosphokinase (CPK), aminotransferase alanine (ALT), aminotransferase aspartate (AST), and lactic dehydrogenase (LDH)], electroneuromyography tests revealing predominance of proximal myopathy with no neurological pattern, muscle biopsy of bicep arm or muscle vastus lateralis muscle with the presence of inflammatory infiltrate in the perimyosial and perivascular area, and/or perifascicular atrophy. Changes in high-resolution computed tomography images of the lung: incipient interstitial lung disease (ILD), ground-glass opacities, and pulmonary fibrosis in both lung bases.
- Outcomes: Diagnosis of neoplasms, severe infections (defined as an infection that required parenteral therapy or tuberculosis), and deaths.
- Disease activity status on the last medical appointment was defined according to the international consensus guidelines for trials of myositis therapies (proposed by the International Myositis Assessment and Clinical Studies Group - IMACS) [27] as clinical remission (no evidence of disease activity for at least six months without DM treatment), complete clinical response (no evidence of disease activity for at least six months while still receiving myositis therapy), or disease relapse: recurrence of clinical (muscle or skin manifestations) and/or laboratory findings (elevated creatine phosphokinase or aldolase) with no explanation other than disease activity.
- Comorbidities: systemic arterial hypertension, type 2 diabetes mellitus, acute myocardial infarction.
- Habits and addictions: current and previous smoking and/or alcohol use disorder.
- Drug treatment: initial treatment (received in the first year after diagnosis) with intravenous methylprednisolone (IVMP) or intravenous immunoglobulin (IVIg), current dose of glucocorticoids, immunosuppressive, immunomodulatory, or immunobiological drugs.

An analysis of the profile of myositis-specific (specific for myositis) and myositis-associated (associated with myositis and other rheumatological diseases) autoantibodies was performed in the serum samples of these patients, collected during the follow-up, and stored at − 20 °C. Autoantibodies (Jo-1, OJ, EJ, PL-7, PL-12, PM/Scl75, PM/Scl100, Ku, SRP, Mi-2α, Mi-β, Ro-52, MDA-5, TIF-1γ, SAE, and NXP-2) were analyzed using an immunoblotting commercial kit (DL 1530-1601-4G, Euroimmun, Lübeck, Germany).

Statistical analysis. The Shapiro–Wilk test was used to assess the normality of the distribution of continuous variables. Results are presented as mean ± standard deviation or median (interquartile range [IQR] 25–75%) for continuous parameters and number (%) for categorical variables. To compare the differences between patients with and without myositis-specific autoantibodies, we used the Student’s *t*-test for continuous variables with normal distribution and the Mann–Whitney U test for continuous variables with non-normal distribution. For categorical variables, differences were calculated using the chi-squared test; Fisher’s exact test was used when > 20% of cells had an expected count of less than 5 in a 2 × 2 contingency table. We further explored the association between clinical, imaging, and laboratory features and the main autoantibodies using a univariate binary logistic regression to calculate odds ratios (OR) with a 95% confidence interval (CI). A *P *value < 0.05 was considered statistically significant. Analyses were performed using IBM SPSS Statistics for Windows (version 24.0; IBM Corp, Armonk, NY, USA).

## Results

**Fig. 1:**
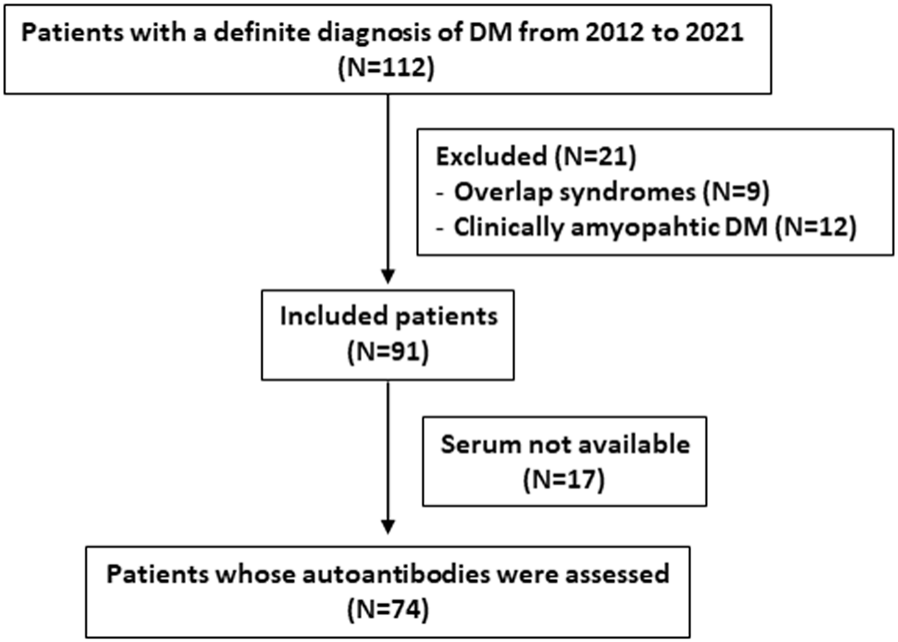
Flowchart of selection of the patients with dermatomyositis from the present study

Of the 112 patients with a defined DM diagnosis who were initially admitted, 21 were excluded (Fig. 1); thus, information was collected from 91 patients, of whom 74 had the autoantibodies analyzed. According to the demographic profile, the mean age at DM diagnosis was 47.3 ± 15.4 years, with a predominance of white females (Table 1).

**Table 1:** General characteristics of 91 adult patients with defined dermatomyositis

Characteristics	N = 91
Age at disease diagnosis (years)	47.3 ± 15.4
Female gender	61 (67.0)
White ethnicity	74 (81.3)
Constitutional symptoms at baseline	81 (89.0)
Gottron’s sign/papules	87 (96.7)
Heliotrope rash	79 (86.8)
“V”-neck sign	71 (78.0)
“Shawl” sign	56 (61.5)
Raynaud’s phenomenon	55 (60.4)
Vasculitis	26 (28.6)
“Mechanics’ hands”	20 (21.0)
Skin ulcers	15 (16.5)
Calcinosis	4 (4.4)
Muscle strength (MRC)	
Upper limbs	
V degree	1 (1.1)
IV degree	61 (67.0)
III degree	23 (25.3)
II degree	6 (6.6)
Lower limbs	
V degree	5 (5.5)
IV degree	56 (61.6)
III degree	24 (26.4)
II degree	6 (6.6)
Dysphagia	67 (73.6)
Arthritis	30 (33.0)
Lung involvement	34 (37.4)
Interstitial pneumopathy	32 (35.2)
Ground glass opacities	12 (13.2)
Pneumomediastinum	1 (1.1)
Maximum levels of muscle enzymes	
Creatine phosphokinase (U/L)	812 (177–3121)
Aspartate aminotransferase (U/L)	62 (26–32)
Alanine aminotransferase (U/L)	43 (21–98)
Lactic dehydrogenase (U/L)	531 (248–904)
Antinuclear antibodies	62 (68.2)
Myositis-specific autoantibodies (N = 74)	
Anti-Mi-2	10 (11.0)
Anti-Jo-1	10 (11.0)
Anti-MDA-5	10 (11.0)
Anti-TIF-1γ	7 (7.7)
Anti-SAE	6 (6.6)
Anti-NXP-2	5 (5.5)
Myositis-associated autoantibodies (N = 74)	
Anti-Ro-52	26 (28.6)
Anti-PM/Scl 75	3 (3.3)
Anti-SRP	1 (1.1)
Anti-OJ, -EJ, PL-7, PL-12, -Ku	3 (3.3)

Data are expressed as mean ± standard deviation, median (IQR 25th–75th), or percentage (%)

The main cutaneous manifestation was Gotttron’s sign or papules (96.7%), followed by a heliotrope rash (86.8%) and “V”-neck sign (78%). At the onset of DM diagnosis, 90% of the patients had muscle weakness with MRC grade IV or III. Systematic manifestations, such as dysphagia, arthritis, and lung involvement were observed in 73.6%, 33.0%, and 37.4%, respectively. Maximum levels of CPK (3121 U/L), AST (32 U/L), ALT (98 U/L), and LDH (904 U/L) were detected in laboratory exams. The full description of general characteristics is summarized in Table 1. The median follow-up time was 44 months (range 17–67 months) (Table 2).

**Table 2:** Treatment characteristics and follow-up of 91 patients with dermatomyositis

Characteristics	N = 91
Current follow-up	75 (83.3)
Duration of follow-up (months)	44 (17–67)
Disease status at the last medical evaluation	
Active disease (clinical relapse)	30 (33.0)
Complete clinical response	44 (48.4)
Disease remission	17 (18.7)
Treatment	
Previous IVMP pulse therapy	70 (76.9)
Previous IVIg	54 (59.3)
Previous IVIg + IVMP pulse therapy	50 (54.9)
No IVMP or IVIg	14 (15.4)
Current treatment	
Glucocorticoid	
Current use	30 (33.0)
Current dose (prednisone equivalent), mg/day	0.0 (0.0–7.5)
Immunosuppressive/immunomodulatory/immunobiological	
Methotrexate	21 (23.1)
Azathioprine	12 (13.2)
Mycophenolate mofetil	12 (13.2)
Leflunomide	8 (8.8)
Cyclosporine	3 (3.3)
Cyclophosphamide	1 (1.1)
Rituximab	30 (33.0)

IVIg, intravenous immunoglobulin; IVMP, intravenous methylprednisolone

Data are expressed as mean ± standard deviation, median (IQR 25th–75th), or percentage (%)

Concerning the initial medical treatment, 76.9% of the patients received IVMP, 59.3% received IVIg, and 54.9% received both medications. During follow-up, patients received different types of immunosuppressants, immunomodulators, or immunobiological, such as methotrexate (23.1%), azathioprine (13.2%), mycophenolate mofetil (13.2%), and leflunomide (8.8%); one-third of the patients received rituximab (Table 2).

Patients’ attendance at regular follow-up was 83.3% (Table 2). Based on the last medical appointment, one-third of the patients had disease relapse, half had a complete clinical response, and one-fifth were in remission. Only one-third of the patients were still on glucocorticoids, with a median dose of 0.0 (IQR 0.0–7.5) mg/day.

There were 92 episodes of severe infections; during the follow-up, the most frequent was community-acquired pneumonia, followed by herpes zoster, tuberculosis, and COVID-19. Twenty patients (22%) developed neoplasms, with the female breasts as the main primary site. Nine patients died of different causes, ranging from infectious (2.2%) to neoplastic processes (4.4%) (Table 3).

**Table 3:** Outcomes during follow-up

Outcomes	N = 91
Cancer	20 (22.0)
Breast	8 (8.8)
Stomach	4 (4.4)
Lung	2 (2.2)
Cervical	1 (1.1)
Uterus	1 (1.1)
Melanoma	1 (1.1)
Thyroid	1 (1.1)
Non-Hodgkin’s lymphoma	2 (2.2)
Nasopharyngeal	1 (1.1)
Gastrointestinal stromal tumors	1 (1.1)
Severe infections	48 (52.7)
Pneumonia	15 (16.5)
Herpes-zoster	11 (12.1)
Tuberculosis	5 (5.5)
COVID-19	3 (3.3)
Other causes	58 (63.7)
Total episodes of infections	92
Death	9 (9.9)
Infections	2 (2.2)
Neoplasia	4 (4.4)
Other causes	3 (3.3)

Data expressed as a percentage (%)

Systemic arterial hypertension was identified in 46.2% of the patients, while diabetes mellitus and acute myocardial infarction were present in 19.8% and 4.4% of the patients, respectively. Former and current smokers were observed in 12.2% and 9.9% of patients, respectively, while former alcohol use disorder was observed in 8.8%; current alcohol use disorder was present in only one patient.

The clinical, imaging, and laboratory features among the main autoantibodies profiles are shown in Table 3. A univariate binary logistic regression (Table 4) has shown that heliotrope rash and Raynaud’s phenomenon were associated with positive antinuclear antibodies (ANA) (OR = 3.63, 95%CI 1.04–12.64, *P* = 0.043, and OR = 2.58, 95%CI 1.05–6.39, *P* = 0.040, respectively). “V”-neck sign (OR = 0.07, 95%CI 0.02–0.28, *P* < 0.001) and “shawl” sign (OR = 0.22, 95%CI 0.06–0.79, *P* = 0.021) had an inverse association with anti-tRNA synthetases, while “mechanics’ hands” (OR = 5.30, 95%CI 1.49–18.90, *P* = 0.010), ILD (OR = 5.38, 95%CI 1.47–19.72, *P* = 0.011), and anti-Ro-52 antibodies (OR = 3.82, 95%CI 1.10–13.28, *P* = 0.035) had a positive association with anti-tRNA synthetases. Male gender (OR = 4.90, 95%CI 1.22–19.69, *P* = 0.025), digital ulcers (OR = 9.67, 95%CI 2.16–43.22, *P* = 0.003), vasculitis (OR = 12.00, 95%CI 2.31–62.46, p = 0.003), arthritis (OR = 4.78, 95%CI 1.12–20.36, *P* = 0.034), anti-Ro-52 (OR = 5.53, 95%CI 1.29–23.68, *P* = 0.021), and active disease (OR = 6.11, 95%CI 1.47–25.43, *P* = 0.013) were associated with anti-MDA-5 antibodies. “Mechanics’ hands” (OR = 4.30, 95%CI 1.41–13.13, *P* = 0.011), arthritis (OR = 3.67, 95%CI 1.34–10.03, *P* = 0.011), RP-ILD (OR = 3.82, 95%CI 1.10–13.28, *P* = 0.035), anti-tRNA synthetases (OR = 3.82, 95%CI 1.10–13.28, *P* = 0.035), and anti-MDA-5 (OR = 5.53, 95%CI 1.29–23.68, *P* = 0.021) were associated with anti-Ro-52 antibodies (Table 5).

**Table 4:** Comparison of clinical, imaging, and laboratory features among different autoantibodies profiles in patients with dermatomyositis

Characteristics	ANA	Anti-ARS	Anti-MDA-5	Anti-Mi-2	Anti-Ro-52
	(n = 62)	(n = 13)	(n = 10)	(n = 10)	(n = 26)
Age at diagnosis (years)	47.9 (16.1)	47.2 (11.1)^d^	45.8 (17.2)	50.5 (12.6)^d^	49 (13.9)
Female gender	40 (64.5)	9 (69.2)^e^	4 (40.0)^a,e^	8 (80.0)^e^	16 (61.5)
White ethnicity	49 (79.0)	11 (84.6)^e^	7 (70.0)^e^	8 (80.0)^e^	19 (73.1)
Fever/weight loss	56 (90.3)^e^	12 (92.3)^e^	10 (100)^e^	10 (100)^e^	25 (96.2)^e^
Gottron’s sign	61 (98.4)^e^	12 (92.3)^e^	10 (100)^e^	10 (100)^e^	26 (100)^e^
Heliotrope rash	57 (91.9)^a,e^	9 (69.2)^e^	9 (90.0)^e^	7 (70.0)^e^	21 (80.8)
“V”-neck sign	51 (82.3)	5 (38.5)^b,e^	10 (100)^e^	8 (80.0)^e^	20 (76.9)^e^
“Shawl” sign	37 (59.7)	4 (30.8)^a,e^	8 (80.0)^e^	8 (80.0)^e^	14 (53.8)
Raynaud’s phenomenon	42 (67.7)^a^	7 (53.8)^e^	7 (70.0)^e^	7 (70.0)^e^	19 (73.1)
“Mechanics’ hands”	15 (24.2)	7 (53.8)^a,e^	4 (40.0)^e^	0^e^	11 (42.3)^b^
Digital ulcers	13 (21.0)	0^e^	5 (50.0)^b,e^	2 (20.0)^e^	4 (15.4)^e^
Vasculitis	20 (32.3)	1 (7.7)^a,e^	8 (80.0)^b,e^	3 (30.0)^e^	9 (34.6)
Calcinosis	2 (3.2)^e^	0^e^	0^e^	0^e^	0^e^
Muscle weakness	19 (30.6)	3 (23.1)^e^	2 (20.0)^e^	5 (50.0)^e^	5 (19.2)
Dysphagia	45 (72.6)	8 (61.5)^e^	8 (80.0)^e^	7 (70.0)^e^	20 (76.9)
Arthritis	21 (33.9)	8 (61.5)^e^	7 (70.0)^a,e^	1 (10.0)^e^	15 (57.7)^a^
ILD	23 (37.1)	9 (69.2)^b,e^	4 (40.0)^e^	0^a^^,e^	13 (50.0)
RP-ILD	10 (16.1)^e^	4 (30.8)^e^	2 (20.0)^e^	0^e^	8 (30.8)^e^
Maximum CPK (U/L)	1245 (189–3931)^d^	585 (216–5323)^d^	158 (91–857)^a,d^	4783 (3366–8915)^b,d^	301 (117–2781)^d^
ANA	–	8 (61.5)^e^	8 (80.0)^e^	9 (90.0)^e^	18 (69.2)
Anti-ARS	8 (12.9)^c,e^	–	0^e^	0^e^	8 (30.8)^e^
Anti-MDA-5	8 (12.9)^c,e^	0^e^	–	0^e^	7 (26.9)^a,e^
Anti-Mi-2	9 (14.5)^c,e^	0^e^	0^e^	–	0^a^^,e^
Anti-Ro-52	18 (29.0)^c^	8 (61.5)^a,e^	7 (70.0)^a,e^	0^a^^,e^	–
Severe infections	32 (51.6)	5 (38.5)	5 (50.0)^e^	5 (50.0)^e^	12 (46.2)
Neoplasia	12 (19.4)	2 (15.4)^e^	1 (10.0)^e^	0^e^	5 (19.2)
Complete clinical response	31 (50.0)	8 (61.5)	4 (40.0)^e^	6 (60.0)^e^	14 (53.8)
Disease remission	22 (35.5)	4 (30.8)^e^	1 (10.0)^e^	4 (40.0)^e^	7 (26.9)
Active disease	9 (14.5)	1 (7.7)^e^	5 (50.0)^a,e^	0^e^	5 (19.2)^e^
Death	6 (9.7)^e^	2 (15.4)^e^	0^e^	0^e^	3 (11.5)^e^

ANA, antinuclear antibody; ARS, anti-tRNA synthetase; CPK, creatine phosphokinase; ILD, interstitial lung disease; RP, rapidly progressive

Data are expressed as a percentage (%) or median (IQR 25–75). To determine differences between patients with and without the specific autoantibody, Student’s t-test was used for continuous variables, unless otherwise stated; for categorical variables, differences were calculated using the chi-squared test, unless otherwise stated

^a^*P* < 0.05

^b^*P* < 0.01

^c^Data available from 52 patients

^d^Mann–Whitney U test

^e^Fisher’s exact test

**Table 5:** Autoantibodies and their association with specific sets of clinical, imaging, and laboratory features of dermatomyositis using a univariate binary logistic regression

Characteristics	ANA (n = 62)		Anti-ARS (n = 13)		Anti-MDA-5 (n = 10)		Anti-Ro-52 (n = 26)	
	*P*	OR (95% CI)	*P*	OR (95% CI)	*P*	OR (95% CI)	*P*	OR (95% CI)
Male gender	NS	NS	NS	NS	0.025	4.90 (1.22–19.69)	NS	NS
Heliotrope rash	0.043	3.63 (1.04–12.64)	NS	NS	NS	NS	NS	NS
“V”-neck sign	NS	NS	< 0.001	0.07 (0.02–0.28)	NS	NS	NS	NS
“Shawl” sign	NS	NS	0.021	0.22 (0.06–0.79)	NS	NS	NS	NS
Raynaud’s phenomenon	0.040	2.58 (1.05–6.39)	NS	NS	NS	NS	NS	NS
“Mechanics’ hands”	NS	NS	0.010	5.30 (1.49–18.90)	NS	NS	0.011	4.30 (1.41–13.13)
Digital ulcers	NS	NS	NS	NS	0.003	9.67 (2.16–43.22)	NS	NS
Vasculitis	NS	NS	NS	NS	0.003	12.00 (2.31–62.46)	NS	NS
Arthritis	NS	NS	NS	NS	0.034	4.78 (1.12–20.36)	0.011	3.67 (1.34–10.03)
ILD	NS	NS	0.011	5.38 (1.47–19.72)	NS	NS	NS	NS
RP-ILD	NS	NS	NS	NS	NS	NS	0.035	3.82 (1.10–13.28)
Anti-ARS	NS	NS	–	–	NS	NS	0.035	3.82 (1.10–13.28)
Anti-MDA-5	NS	NS	NS	NS	–	–	0.021	5.53 (1.29–23.68)
Anti-Ro-52	NS	NS	0.035	3.82 (1.10–13.28)	0.021	5.53 (1.29–23.68)	NS	NS
Active disease	NS	NS	NS	NS	0.013	6.11 (1.47–25.43)	NS	NS

No association was found between anti-Mi-2 and the several clinical, imaging, and laboratory characteristics assessed

The following characteristics had no statistical association with specific autoantibodies and therefore were not included in the table: age at diagnosis (years), ethnicity, fever/weight loss, Gottron’s sign or papules, calcinosis, muscle weakness, dysphagia, maximum CPK, ANA, anti-Mi-2, severe infections, neoplasia, complete clinical response, disease remission, and death

ANA, antinuclear antibody; ARS, anti-tRNA synthetase; CPK, creatine phosphokinase; ILD, interstitial lung disease; NS, not significant; *P*, two-tailed *P*-value; RP, rapidly progressive

## Discussion

This study evaluated the demographic, clinical, laboratory, therapeutic, and outcome characteristics of patients with DM, during a median follow-up time of 44 months. The frequency of clinical visits could not be established, since those were individualized based on the severity of the clinical condition, disease duration, presence of extramuscular characteristics, and the treatment performed.

There are only a few epidemiological studies of patients with DM [6–23]; many of them are limited to cross-sectional [7, 9, 11, 16, 17] or retrospective studies [6, 12, 14, 15, 18, 20, 22] with a small [13, 18, 23] or heterogeneous sample of patients with DM [9, 11, 13, 18, 23]. Of these, only five studies specifically evaluated a cohort of Brazilian patients [6, 7, 14, 15, 17]. A list of previous epidemiological studies in patients with DM and a comparison with our findings is summarized in Additional file 1: Table S1 and discussed in the following paragraphs.

In contrast to the articles mentioned above, the present study analyzed a longitudinal database, thus reducing methodological biases. Strict inclusion and exclusion criteria were applied to eliminate possible confounding factors. Finally, to characterize myositis-specific and myositis-associated autoantibodies, a commercial kit was used.

Patients with DM usually present skin involvement followed by muscle weakness [28]. Regarding the classic cutaneous signs, heliotrope and Gottron’s papules/sign are the most closely linked to DM [6, 7, 12, 14, 15, 17, 19, 20]. Likewise, our study revealed that the most common skin finding was Gottron’s papules/sign.

Other skin abnormalities, such as calcinosis, are associated with impaired quality of life due to ulcerations and secondary infections, which develop in approximately 30% of adult patients with DM [29]. In contrast to the available literature, this manifestation was much lower (4.4%) in our sample, possibly due to early diagnosis and aggressive initial treatment.

Excluding the primary involvement sites of DM, the lungs are the most affected sites, and manifestations such as ILD are associated with higher morbidity and mortality [7, 14, 17, 30]. A meta-analysis by Sun et al. [29] showed a prevalence of 41% of ILD in patients with DM, predominantly among Asians. In the present study, 37.4% of the patients had pulmonary involvement and 35.2% had ILD.

Gastrointestinal manifestations are well known in DM, and the main symptom is oropharyngeal dysphagia. The severe form of dysphagia has a wide prevalence, ranging from 10 to 73% [31]. Corroborating data from the literature, the prevalence of dysphagia in our study was 73.6%.

The treatment approach should be individualized based on the severity of clinical presentation, disease duration, presence of extramuscular characteristics, prior therapies, and contraindications to specific agents [32, 33]. The therapeutic regimen implemented in our service initially comprised the administration of oral glucocorticoids at a dose of 0.5–1.0 mg/kg/day. Subsequently, immunosuppressants and immunomodulators were indicated for the most severe cases. In addition to these medications, other early interventions (during the first year) were performed using IVMP, IVIg, or both.

Previous studies have shown an increase in patient survival or higher rates of complete clinical response when glucocorticoids were administered earlier and at high doses [32, 33]. In addition, an early approach to targeted treatment with IVMP and/or IVIg was associated with a potential reduction in long-term muscle disability and better outcomes (complete clinical response and discontinuation of corticosteroids) [34, 35].

It is important to emphasize that adjuvant therapies with immunomodulators and immunosuppressants, such as leflunomide, seem to be safe and effective for cases of refractory DM with primary cutaneous activity [36]. Due to the established therapeutic regimen, it was possible to verify at the last visit, that approximately half of the patients developed complete clinical response and only 27.3% still showed disease activity.

Infections are associated with increased mortality in patients with DM, leading to death in 9–30% of cases. A wide variety of microorganisms may be responsible for pyogenic and opportunistic infections in DM. The most common are mycobacteria and fungi (*Pneumocystis jirovecii*, *Candida* spp.) [37]. The rate of serious infections in DM patients is 11.1 cases/100 patients/year; The main cause is aspiration pneumonia, followed by opportunistic infections [38].

During follow-up, there were 92 episodes of severe infection, the most frequent being community-acquired pneumonia, followed by herpes zoster, tuberculosis, COVID-19, and other infections.

The prevalence of systemic arterial hypertension and diabetes mellitus was high at the onset of myositis and tended to increase after the diagnosis of myositis [39, 40]. Comorbidities, such as systemic arterial hypertension, diabetes mellitus, and acute myocardial infarction, were present in 46.2%, 19.8%, and 4.4% of patients, respectively. The high prevalence of systemic arterial hypertension in the present study was only observed in previous studies that presented heterogeneous patient samples (with different inflammatory myopathies) [40].

Malignant neoplasms have been associated with DM, including gynecological ones, particularly ovarian carcinoma [23]. Compared to the literature, this study revealed a higher occurrence of cancer; the principal primary site was the female breast [12, 23]. This finding could be explained by the fact that these articles only considered cases of cancer with an interval no more than three years before or after the DM diagnosis. In addition, the follow-up periods in these studies were shorter than those presented herein (44 months).

This study also assessed the correlation between autoantibodies and patient characteristics and outcomes. The main autoantibodies were observed at a similar frequency in comparison with other studies [6, 7, 9, 12–14, 18, 20–23]: anti-Mi-2 (11%), anti-MDA-5 (11%), anti-Jo-1 (11%), and anti-Ro-52 (28.6%). The different frequencies of autoantibodies among studies may be explained by the small and heterogeneous samples of patients and distinct assessment methods. A strength of our study is that we evaluated a homogeneous population and used an accurate autoantibody analysis assay.

According to the literature, anti-Jo-1 is associated with joint involvement, “mechanic’s hands”, and ILD [15]; anti-Mi-2 is associated with cutaneous manifestations, low frequency of pulmonary involvement, low glucocorticoid requirement, and high DM remission rate [14]; and anti-MDA-5 is associated with different forms of skin involvement, especially skin ulcers and others resembling antisynthetase syndrome [6, 7, 18, 19]. Similarly, the associations found were anti-tRNA synthetases with ILD, “mechanic’s hands”, and anti-Ro52, with a negative association with “V”-neck and “shawl” signs. Anti-MDA-5 were associated with male gender, digital ulcers, vasculitis, arthritis, anti-Ro-52, and active disease. Anti-Ro-52 were associated with “mechanics’ hands”, arthritis, rapidly-progressive ILD, anti-tRNA synthetases, and anti-MDA-5. Contrary to other studies, no correlation was observed between anti-Mi-2 and cutaneous manifestations, frequency of pulmonary involvement, glucocorticoid use, or disease remission.

As limitations of this study, osteoporosis, opportunistic infections, disability, and quality of life were not evaluated. Another limitation was related to Muscle strength graded classification, as we used MRC instead of MMT-8.

## Conclusions

The present study demonstrated the clinical, laboratory, and outcome characteristics of Brazilians with a definitive diagnosis of DM and explored the associations between myositis-specific and myositis-associated DM autoantibodies in this homogeneous population. Therefore, our thorough data allowed better characterization of DM in terms of clinical manifestations, evolution, and prognosis in DM.

## Supplementary Information

**Additional file 1. Complementary Table 1**. Epidemiological studies in patients with dermatomyositis.

## Data Availability

Not applicable.

## Abbreviations

ACR: American College of Rheumatology
ALT: Aminotransferase alanine
ANA: Antinuclear antibody
AST: Aminotransferase aspartate
CPK: Creatine phosphokinase
EULAR: European League Against Rheumatism
IQR: Interquartile range
ILD: Incipient interstitial lung disease
IVMP: Intravenous methylprednisolone
IVIg: Intravenous immunoglobulin
LDH: Lactic dehydrogenase
MRC: Medical Research Council
