# Supplementary material for: Clinical manifestations, outcomes, and antibody profile of Brazilian adult patients with dermatomyositis: a single-center longitudinal study
Source: Adv Rheumatol. 2022 Nov 12;62(1):41. doi: 10.1186/s42358-022-00276-x (PMC9660096; doi:10.1186/s42358-022-00276-x)
Supplement: Supplementary file 1 — Additional file 1. Complementary Table 1. Epidemiological studies in patients with dermatomyositis. [file 42358_2022_276_MOESM1_ESM.docx]

**Supplementary Table 1. Epidemiological studies in patients with dermatomyositis**


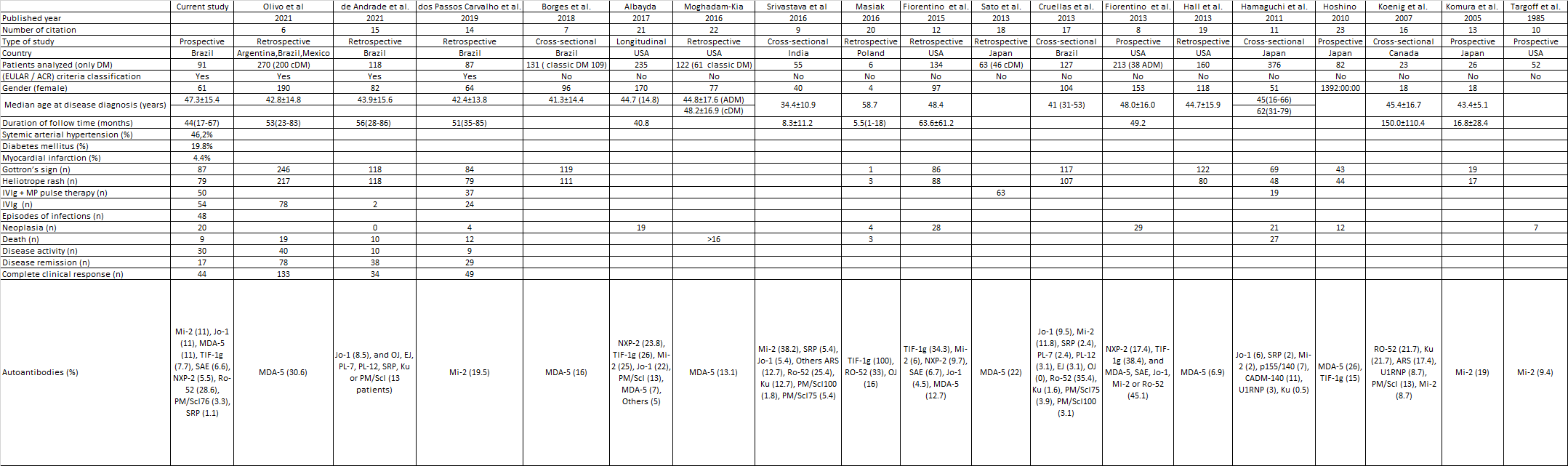


ADM: amyopathic dermatomyositis; ARS: aminoacy-tRNA synthetases; cDM: classical dermatomyositis; DM: dermatomyositis; IVIg: intravenous human immunoglobulin; MP: methylprednisolone.
